# Candidate loci for the kernel row number in maize revealed by a combination of transcriptome analysis and regional association mapping

**DOI:** 10.1186/s12870-019-1811-1

**Published:** 2019-05-16

**Authors:** Yixin An, Lin Chen, Yong-Xiang Li, Chunhui Li, Yunsu Shi, Yanchun Song, Dengfeng Zhang, Yu Li, Tianyu Wang

**Affiliations:** Institute of Crop Sciences, Chinese Academy of Agricultural Sciences, Beijing, 100081 China

**Keywords:** Maize, Inflorescence development, Kernel row number (KRN), Introgression lines, Regional association mapping

## Abstract

**Background:**

The kernel row number (KRN) of an ear is an important trait related to yield and domestication in maize. Exploring the underlying genetic mechanisms of KRN has great research significance and application potential.

**Results:**

In the present study, N531 with a KRN of 18–22 and SLN with a KRN of 4–6 were used as the recurrent parent and the donor parent, respectively, to develop two introgression lines (ILs), IL_A and IL_B, both of which have common negative-effect alleles from SLN on chromosomes 1, 5 and 10 and significantly reduced inflorescence meristem (IM) diameter and KRN compared with those of N531. We used RNA-Seq to investigate the transcriptome profiles of 5-mm immature ears of N531, IL_A and IL_B. We identified a total of 2872 differentially expressed genes (DEGs) between N531 and IL_A, 2428 DEGs between N531 and IL_B and 1811 DEGs between IL_A and IL_B. A total of 1252 DEGs were detected as overlapping DEGs, and 89 DEGs were located on the common introgression fragments. Furthermore, three DEGs (Zm00001d013277, Zm00001d015310 and Zm00001d015377) containing three SNPs associated with KRN were identified using regional association mapping.

**Conclusions:**

These results will facilitate our understanding of ear development and provide important candidate genes for further study on KRN.

**Electronic supplementary material:**

The online version of this article (10.1186/s12870-019-1811-1) contains supplementary material, which is available to authorized users.

## Background

Maize (*Zea mays*) is widely grown worldwide and is one of the most important food and feed crops with important economic significance [1]. As an important yield component, the kernel row number (KRN) has a significantly positive effect on yield [2, 3]. In addition, KRN is a typical domestication trait that varied greatly from teosintes to modern maize [2, 4]. Thus, exploring the genetic basis of KRN and identifying functional genes are necessary to provide new insights into yield improvement and the domestication of maize.

The formation of KRN involves a number of meristem initiations, determinacies and identities during the development of the ear [5]. Maize forms inflorescence meristems (IMs) from the vegetative phase to the reproductive phase. IMs further differentiate and develop into spikelet pair meristems (SPMs). Even spikelet meristems (SMs) eventually form KRN through the process of floral meristem (FM) development and pollination [6]. In summary, four types of meristems with different identities and fates (IMs, SPMs, SMs and FMs) eventually form the mature ear of maize [5, 6]. Although the formation of KRN seems to be rather simple, the molecular mechanism is relatively complex in maize and remains obscure.

To fully understand the developmental characteristics of female inflorescence and KRN, substantial effort has been undertaken to identify causative genes through linkage analysis, association analysis and mutant analysis. To date, many quantitative trait loci (QTLs) related to KRN have been identified in different genetic populations of maize (https://www.maizegdb.org) [7]. However, only one QTL, *KRN4,* was cloned [8]. Conversely, many genes involved in the *RAMOSA* pathway (this pathway contains three important genes, *RAMOSA1*, *RAMOSA2* and *RAMOSA3*, which are key regulators of the determinacy of branch meristems and spikelet pair meristems), the CLAVATA-WUSCHEL (CLV-WUS) negative feedback loop (which is a key pathway regulating SAM proliferation and differentiation in Arabidopsis and is widely conserved in other species) and phytohormone signals were identified through genetic assays of inflorescence mutants [9–20]. Additionally, transcription factors (TFs) also play important roles in KRN formation, and several studies have shown that *unbranched2* (*ub2*) and *unbranched3* (*ub3*), as SQUAMOSA Promoter-Binding Protein (SBP) TFs, affect tassel branch number and KRN by limiting the rate of cell differentiation to the lateral domains of meristems [19, 20]. Given the above, although much progress has been made in characterizing the development of female inflorescence and KRN, its regulation remains to be elucidated.

More recently, whole transcriptome RNA sequencing (RNA-seq) and its subsequent transcriptome analyses have been used in many studies to discover candidate genes and pathways responsive to target traits in several plant species. For example, Xiao et al. [21] provided potential candidate genes for *qHS3*, which is a QTL controlling starch content in maize, and identified the coexpression network caused by *qHS3* in maize kernels through transcriptome analysis of near-isogenic lines (NILs). In wheat, Xiao et al. [22] identified some genes involved in *Fusarium* head blight (FHB) resistance and related pathways mediated by *Fhb1* in the wheat landrace “Wangshuibai” based on transcriptome analysis. In rice, Yang et al. [23] compared the transcriptomes of the ovules of a high-frequency female-sterile line (*fsv1*) and a rice wild-type line (Gui 99) and identified a large number of differentially expressed genes (DEGs), including TF genes and epigenetic-related genes, which might play roles in ovule development and fertile female gametophyte formation. Therefore, the high accuracy and sensitivity of RNA-Seq make it a powerful technology for detecting important genes related to interesting traits. In addition, the combination of linkage mapping and association mapping has recently proven to be an effective method to identify candidate loci related to yield-related traits in maize. For example, *qGW4.05*, *qKL1.07* and *qKW7.02* were mapped to larger genomic regions through linkage mapping, and their intervals were further narrowed down by regional association mapping to discover causal genes [24–26]. Three potential candidate genes for kernel size and weight within three MQTL regions in maize were successfully identified based on the combination of MetaQTL analysis and regional association mapping [27].

In this study, we selected two introgression lines (ILs, IL_A and IL_B) and their recurrent parent, N531, which vary in KRN to perform RNA-Seq of the 5-mm immature female ears. The expression profiles of genes were analyzed to identify DEGs. Then, regional association mapping was applied to detect candidate DEGs combining the results of RNA-Seq. Our results will provide important clues and foundations for understanding inflorescence development and KRN formation in maize.

## Results

### Phenotypic and genotypic analysis of N531, IL_A and IL_B

**Fig. 1:**
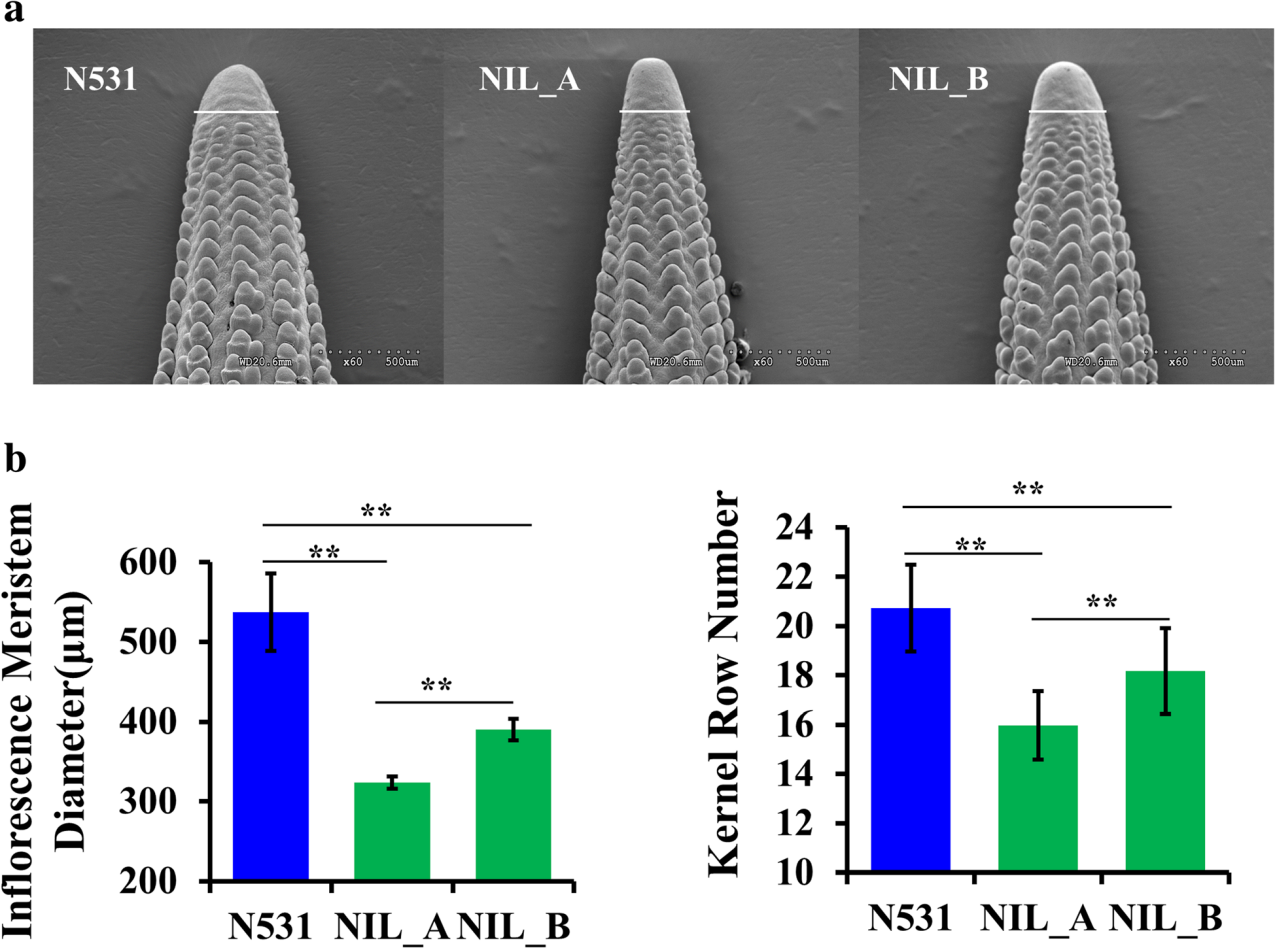
Phenotypic performance of N531, IL_A and IL_B. **a** Micrograph of 5-mm immature ears of N531, IL_A and IL_B, imaged with a scanning electron microscope (SEM). White lines represent inflorescence meristem diameters. Bar = 500 μm. **b** Statistical analysis of inflorescence meristem diameters among N531, IL_A and IL_B. **c** Statistical analysis of kernel row number among N531, IL_A and IL_B. *P*-values were estimated based on the two-tailed Student’s *t*-test, * *P* < 0.05, ** *P* < 0.01, *N* = 9

A major KRN QTL on chromosome 5 (Chr. 5), *qKRN5.04*, was identified in the F_2:3_ and backcross populations in our previous study [28, 29]. Then, IL_A and IL_B carrying the low KRN allele of *qKRN5.04* were selected in the BC_5_F_7_. Comparing their phenotypes, we found that the IM size of the 5-mm immature ear was significantly different among N531, IL_A and IL_B (Fig. 1a and b). The diameter of IM in N531 was larger than that of IL_A (*P*-value = 0.004) and IL_B (*P*-value = 0.015), and the diameter of IM in IL_B was larger than that of IL_A (*P*-value = 0.004). In the mature ear, N531 displayed more than 20 rows (20.7 ± 1.7), which was higher than IL_A (15.9 ± 1.3, *P*-value = 2.70 × 10^− 21^) and IL_B (18.1 ± 1.7, *P*-value = 2.42 × 10^− 6^), and IL_B exhibited a higher KRN than IL_A (*P*-value = 8.95 × 10^− 10^, Fig. 1c).

**Fig. 2:**
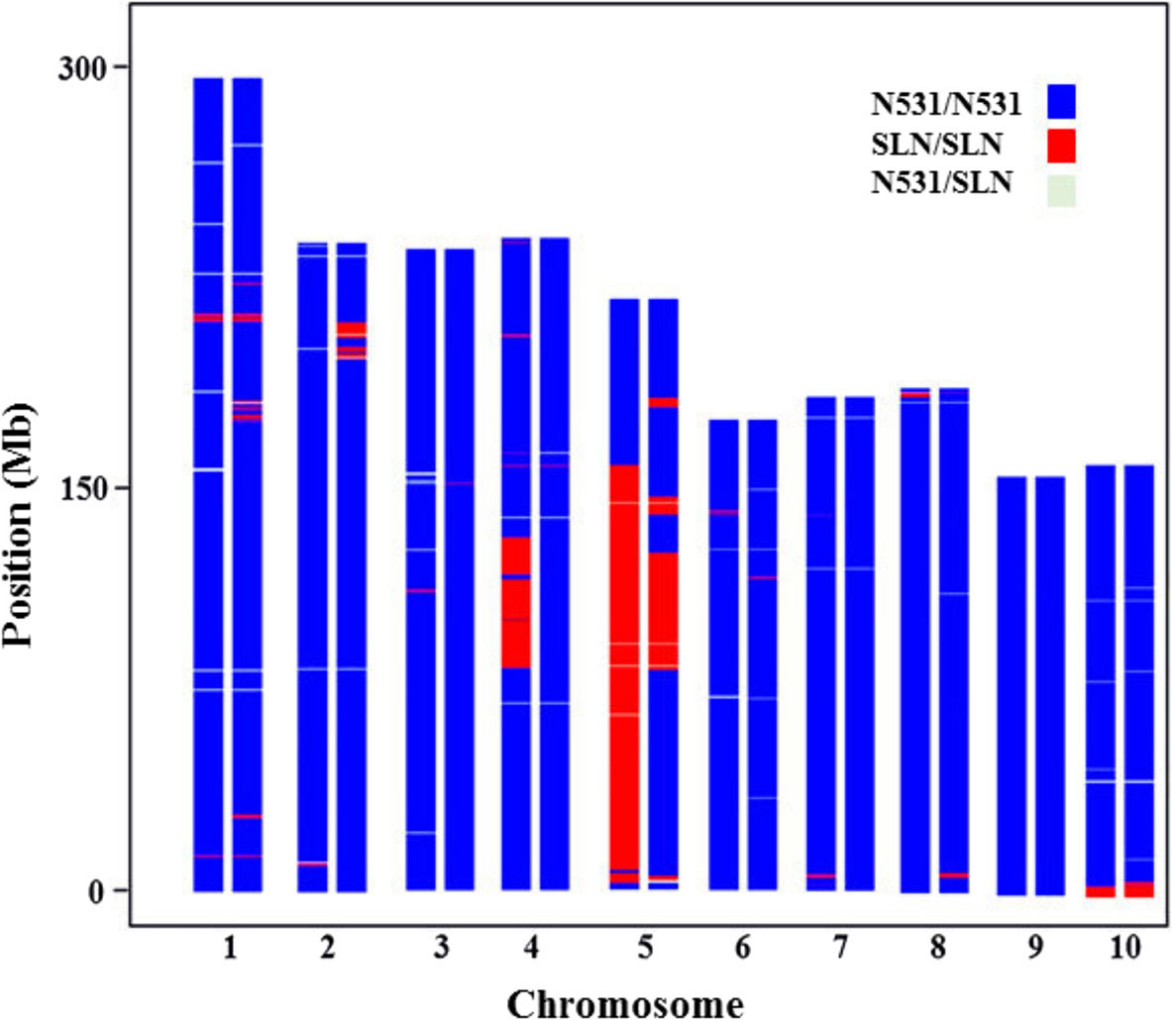
Genomic composition of IL_A (left) and IL_B (right). Blue represents regions homozygous with N531; red represents regions homozygous with SLN; and light green represents regions heterozygous regions

To evaluate the genetic background and donor fragment of the ILs, we genotyped IL_A and IL_B together with N531 and SLN using 56,110 SNPs derived from the MaizeSNP50 BeadChip. A total of 12,099 high-quality SNPs existed between the parents, with the varied number of SNPs on different chromosomes ranging from 835 (Chr. 10) to 1760 (Chr. 1, Additional file 8: Table S1). Most chromosomes of IL_A shared 97–99% of the genotypes of N531, except Chr. 4 (88%) and Chr. 5 (35%), while IL_B shared 94–99% identical genetic background with the recurrent parent N531 except Chr. 5 (80%, Additional file 8: Table S1, Fig. 2).

### Sequencing and mapping results of RNA-Seq

We sampled 5-mm immature ears of N531, IL_A and IL_B to construct cDNA libraries and perform transcriptome sequencing. After filtering and quality control of the raw reads obtained by RNA-Seq, a total of 307,355,502 clean reads were obtained from all libraries, with reads for each sample ranging from 44,351,818 to 59,763,832 (average = 51,225,917, Additional file 9: Table S2). Approximately 83% of these reads were mapped to the B73 genome, of which 96% (248,264,351/256,019,590) were uniquely mapped (Additional file 9: Table S2). Genome-wide gene expression was estimated with FPKM (expected number of Fragments Per Kilobase of transcript sequence per Millions base pairs sequenced), and the correlation analysis results revealed a high Pearson’s correlation coefficient between biological replicates (> 0.98) for all samples analyzed, indicating high reproducibility of the sequencing data (Additional file 1: Figure S1).

### Identification and analysis of DEGs

**Fig. 3:**
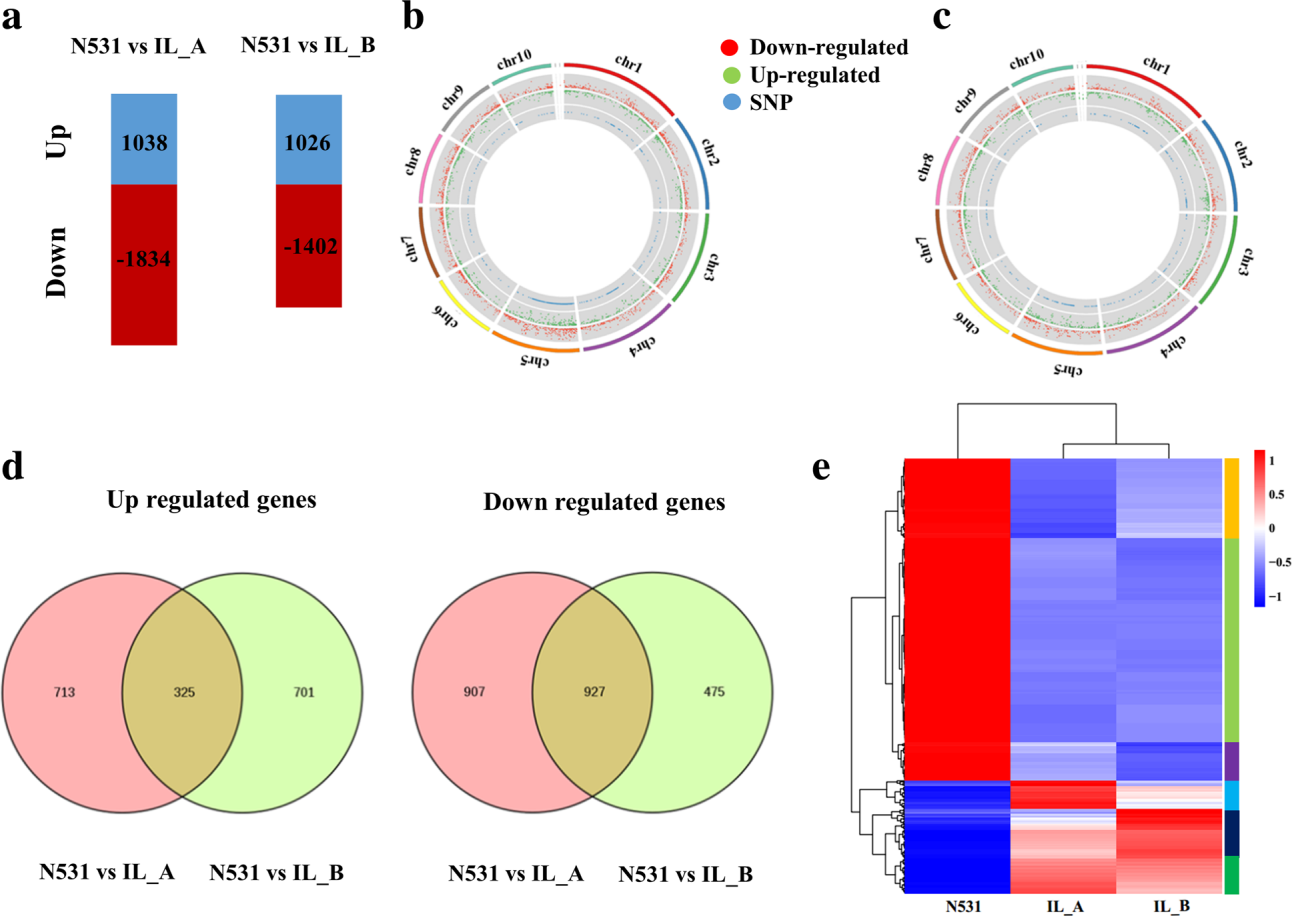
Transcriptome analysis of N531 and ILs. **a** Number of upregulated genes (blue) and downregulated genes (red) between N531 and IL_A, N531 and IL_B. **b**-**c** Distribution of single nucleotide polymorphisms (SNPs) and differentially expressed genes (DEGs) at the 5-mm immature ear stage between N531 and IL_A (b), N531 and IL_B **c** The three circles from inner to outer show the distribution of SNPs (blue dots), upregulated genes (green dots) and downregulated genes (red dots), respectively. **d** Venn diagrams showing the overlap of DEGs (upregulated and downregulated genes) between N531 and ILs. **e** Heatmap clustering 1252 common DEGs by their expression abundance (FPKM). The different colors represent different levels of FPKM. The rectangular bar consisted of 6 colors representing different clusters from I to VI

To identify important genes responsible for KRN variation via transcriptional regulation, we first identified DEGs within two comparison groups: N531 vs IL_A and N531 vs IL_B. In total, 1038 and 1026 DEGs were identified as upregulated genes and 1834 and 1402 DEGs were downregulated genes in the two groups, respectively (Fig. 3a, Additional file 10: Tables S3 and Additional file 11: S4). Between N531 and IL_A, the 2872 (1038 + 1834) DEGs are distributed throughout the maize genome, although there are more SNPs distributed in the introgression fragments on Chr. 4 and Chr. 5 (Fig. 3b). A similar distribution of SNPs and DEGs was found in N531 vs IL_B (Fig. 3c). Among all the DEGs, 1252 genes were common DEGs that were in the same direction between the two groups, including 325 upregulated genes and 927 downregulated genes (Fig. 3d, Additional file 12: Table S5). Heat map clustering showed an overview of the expression of the DEGs with six patterns (Fig. 3e). In Cluster I, the expression level of downregulated DEGs was different between the ILs and lower in IL_A. The opposite trend was observed in Cluster III. The DEGs in Cluster II showed a similar degree of reduction. The remaining three clusters are the upregulated DEGs with various degrees of difference.

Then, we also identified 1811 DEGs between IL_A and IL_B. Among these genes, 728 genes were upregulated and 1083 genes were downregulated in IL_A (Additional file 13: Table S6). Similar to the DEGs among N531 and the ILs, the DEGs between IL_A and IL_B were distributed throughout the whole maize genome. A total of 53 genes were differentially expressed in the 5-mm immature ear of the three materials, of which 26 were upregulated and 27 were downregulated in N531 (Additional file 14: Table S7). Twelve genes among the 53 genes were located on Chr. 4 and Chr. 5, including Zm00001d013130, which encodes a PIF4 transcription factor, and Zm00001d017932, which encodes the MADS-box protein AGL16.

**Fig. 4:**
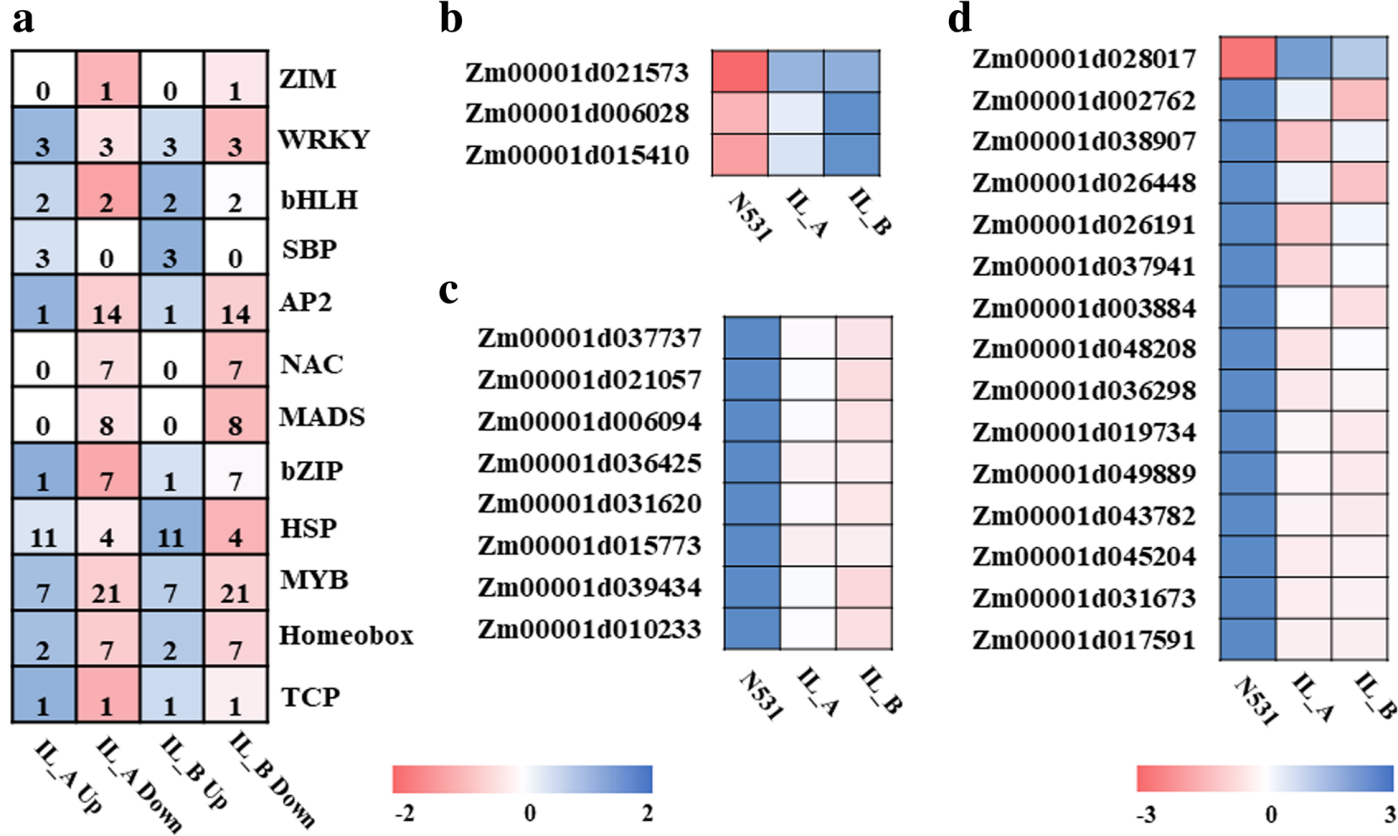
Transcriptome analysis of differentially expressed transcription factors (TFs) between N531 and ILs. **a** Number of differentially expressed TFs. **b** Differentially expressed *SBP* genes. **c** Differentially expressed *MADS-box* genes. **d** Differentially expressed *AP2* genes. The color in each cell indicates the value of FPKM

Among all of the DEGs, many TFs were identified, especially some inflorescence-related TFs, including SBP, MADS-box and AP2 genes (Fig. 4a). Three SBP genes were detected, and they were all significantly upregulated in the ILs (Fig. 4b). Meanwhile, we detected eight MADS-box genes that were all significantly downregulated in the ILs (Fig. 4c). Fifteen AP2 genes were also downregulated, with the exception of Zm00001d028017 (Fig. 4d). The nearly identical expression direction might indicate a consistent inflorescence regulation signal between IL_A and IL_B.

Subsequently, GO analysis was carried out, and the common DEGs were classified into three GO categories: biological process, cellular component and molecular function (Additional file 2: Figure S2a). For biological processes, the three largest categories are photosynthesis (GO: 0015979), photosystem II assembly (GO: 0010207) and photosynthesis, light reaction (GO: 0019684). In the cellular component, the significantly enriched GO terms included chloroplast thylakoid (GO: 0009534), plastid thylakoid (GO: 0031976) and thylakoid (GO: 0009579). Additionally, three major terms, DNA binding (GO: 0003677), nucleic acid binding transcription factor activity (GO: 0001071) and transcription factor activity, sequence-specific DNA binding (GO: 0003700) of molecular function, were also enriched.

The KEGG pathway analysis showed that the common DEGs were significantly enriched in photosynthesis (zma00195), with the highest richness factor and lowest *q* value (Additional file 2: Figure S2b). In addition, other pathways, including protein processing in the endoplasmic reticulum (zma04141), plant hormone signal transduction (zma04075), starch and sucrose metabolism (zma00500) and carotenoid biosynthesis (zma00906), were also markedly enriched in the common DEGs with lower *q* values. The KEGG results can be confirmed by previous studies. For example, *UB3*, which participates in cytokinin biosynthesis and signaling, regulates the kernel row number and the size of the inflorescence meristem in maize and *BIF1*, and *BIF2* regulates inflorescence meristem development via auxin polar transport [16, 17, 19]. Additionally, the *CT2* gene, which has a heterotrimeric G protein α subunit, regulates meristem development through sugar signaling [13]. These results provide useful information for further gene function research in maize. More interestingly, the genes involved in the most enriched GO and KEGG (photosynthesis) term were all downregulated.

### Identification of DEGs responsive to maize inflorescence development

Because common DEGs within the interval of introgression fragments could be used to prioritize candidate causal genes, we identified 89 DEGs located within the common introgression fragments between IL_A and IL_B (Additional file 15: Table S8). On Chr. 1, a known gene *teosinte branched1* (*tb1*, Zm00001d032217), which encodes a member of the TCP transcriptional factor family, was detected [30]. The TB1 protein has pleiotropic effects on plant and inflorescent architecture [31]. In addition, more new genes were identified, including a SBP TF gene on Chr. 5, Zm00001d015410, which was upregulated in the ILs and located in a meta-QTL identified by Chen et al. [27]. Among the 89 DEGs, we found some putative KRN-related genes with large changes in expression. For example, two genes (Zm00001d023265 and Zm00001d015425) were upregulated by more than 500-fold in the ILs. Additionally, we found that ten genes were not expressed in N531 but were expressed in the ILs, whereas 12 genes were expressed in N531 but not in the ILs (Additional file 15: Table S8).

To mine key genes involved in maize inflorescence development, we searched information on Maize Inflorescence (http://www.maizeinflorescence.org/) to obtain the common DEGs’ RNA-Seq expression profiles for the selected set of experiments. We identified 30 genes, including eight upregulated and 22 downregulated genes, which might be involved in maize inflorescence development (Additional file 16: Table S9).

### Regional association mapping

**Fig. 5:**
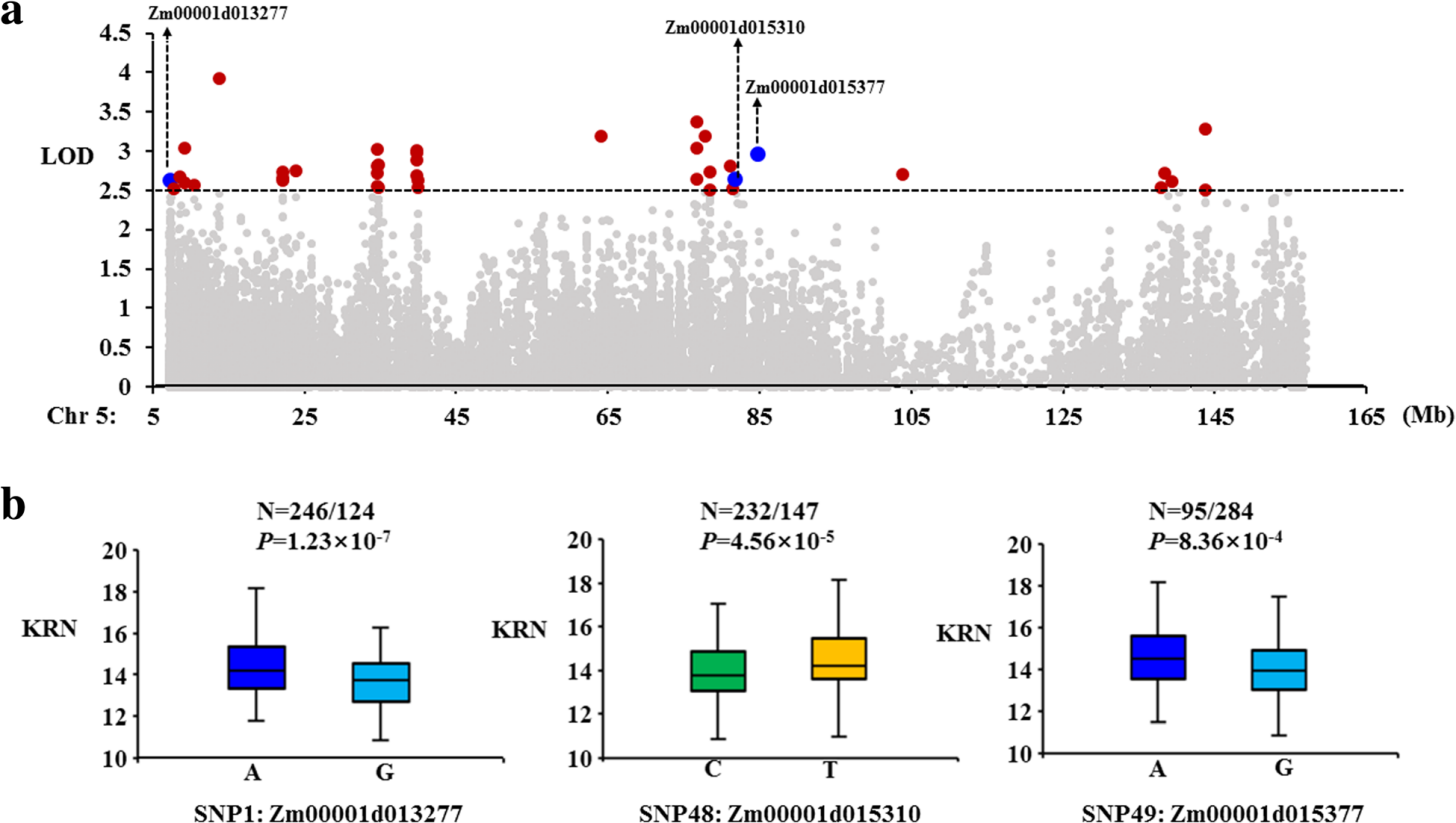
Regional association analysis. **a** Association analysis between the SNPs located in the 7–156 Mb on chromosome 5 and the KRN. The black dashed line represents the significance threshold (LOD > 2.5). The red dots represent the significantly associated SNPs, and the three blue dots represent SNPs located in the DEGs identified by RNA-Seq. **b** Phenotype analysis of maize inbred lines with different alleles of SNP1, SNP48 and SNP49 in the association panel. Boxes with different colors represent different nucleotide bases

We used the strategy of regional association mapping to further analyze the DEGs on Chr. 5. We obtained a total of 28,763 SNPs in an interval of Chr. 5: 7–156 Mb and identified 57 SNPs associated with variation in KRN with a LOD > 2.5 using the MLM method (Fig. 5a). We designated these SNPs SNP1 to SNP57. According to their physical positions on B73 RefGen_v3, we found that these significant SNPs were located in 27 genes (Additional file 17: Table S10). Among these genes, only three genes were identified as DEGs in our study (Fig. 5a). SNP1 was located in the Zm00001d013277 gene (which encodes a retrovirus-related pol polyprotein LINE-1 protein) and had two alleles (A/G) in the association panel, with the A allele being associated with a higher KRN (Fig. 5b, Table 1). For SNP48, which was located in the Zm00001d015310 gene (which encode a cystathionine beta-synthase (CBS) family protein), significant differences in KRN were found between its two alleles (C/T). Previously, we used the RIL population derived from a cross between two elite inbred lines, Huangzaosi (HZS) and LV28, by the single-seed descent method to detect QTLs for yield and its components. *qKRN5–1* was found in Henan in 2009, and this QTL included Zm00001d015310 (Additional file 3: Figure S3) [32]. Zm00001d015377 (which encodes a pentatricopeptide repeat-containing protein) contains SNP49; the A allele of this SNP represented a higher KRN (Fig. 5b, Table 1).

**Table 1:** Candidate genes associated with the kernel row number

Gene ID	Chr.	Position	Gene product	DEGs^a^	Regional association mapping			Meta-QTL region^c^
					SNP number^b^	*R* ^*2*^	Excellent allele	
Zm00001d013277	5	7,694,323–7,698,777	Retrovirus-related pol polyprotein LINE-1 protein	Common DEGs	1	0.03	A	NA
Zm00001d015310	5	84,027,989–84,029,861	A cystathionine beta-synthase (CBS) family protein	Common DEGs	1	0.02	T	MQTL-35
Zm00001d015377	5	87,067,786–87,070,960	A pentatricopeptide repeat-containing (PPR) protein	Common DEGs	1	0.03	A	MQTL-35

a, the gene is the DEG among N531 and the ILs.

b, the number of significant SNPs in the regional association analysis.

c, candidate genes located in the meta-QTL region that were related to the grain yield in the Chen et al. study [27].

### Validation of the identified DEGs by qRT-PCR

To confirm the expression pattern of the DEGs detected by RNA-Seq, real-time quantitative reverse transcription-PCR (qRT-PCR) was performed. Eight genes, including four upregulated and four downregulated genes, were randomly selected from the DEGs located in the common introgression fragments between IL_A and IL_B. As shown in Additional file 4: Figure S4a, the expression trends (up or down) of the eight genes shared high similarity between qRT-PCR and RNA-Seq. The correlation analysis showed a close correspondence (R^2^ > 0.9) in IL_A and IL_B (Additional file 4: Figure S4b).

**Fig. 6:**
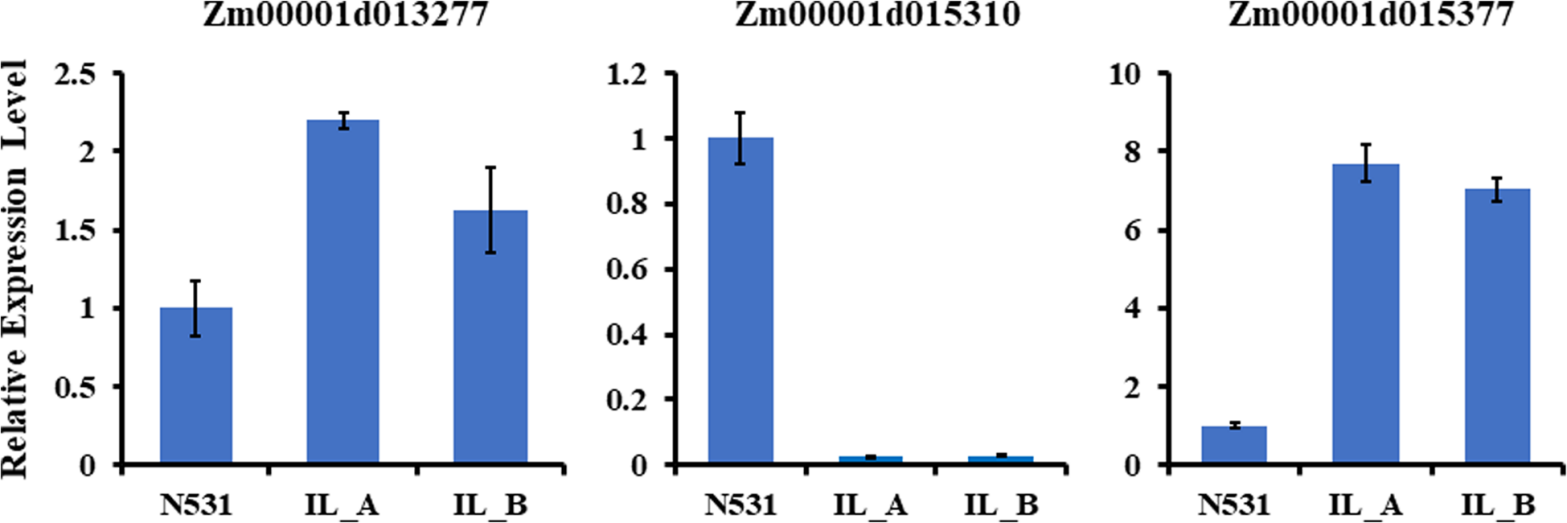
Relative expression levels of three candidate genes in 5-mm immature ears of N531 and the ILs by qRT-PCR

Additionally, three candidate genes were validated with qRT-PCR according to the regional association mapping results. In the 5-mm immature ears, these three genes were indeed differentially expressed between N531 and the ILs (Fig. 6). To test the expression specificity, we also measured their expression levels in different tissues at the three-leaf stage and found that these three genes were also expressed in the root, stem and leaf (Additional file 5: Figure S5).

## Discussion

The IM diameters of IL_A and IL_B were significantly lower than that of N531 at the 5-mm immature female inflorescence, and the KRNs of IL_A and IL_B were also significantly lower than that of N531 at the mature stage. The genotyping results showed that the common introgression fragments shared by IL_A and IL_B were located mainly on chromosomes 1, 5 and 10 in the N531 background. On Chr. 5, both ILs carried the low KRN allele of *qKRN5.04* from SLN. In our previous study, *qKRN5.04,* which accounted for 9.83% of the KRN variation in the F_2:3_ population, was identified, and its effect value was 0.80–1.76 rows in the backcross populations [28, 29]. In the present study, IL_A exhibited smaller IM diameters and KRNs than IL_B, which might result from the larger introgression fragments on chromosomes 4 and 5. These results indicated that those genes for *qKRN5.04* and other introgression fragments might jointly affect KRN through certain pathways.

Recently, RNA-Seq technology was used to identify genes related to many traits in maize, such as starch biosynthesis in maize kernel [21], maize resistance to *Fusarium graminearum* [33], and drought tolerance in maize [34]. In this study, we used RNA-Seq to compare the transcriptomes of 5-mm immature ears between the recurrent parent and its ILs to mine KRN-related genes and pathways. In total, 2872 and 2428 DEGs were identified between N531 and IL_A and between N531 and IL_B, respectively. The higher number of DEGs in IL_A was probably due to its larger introgression fragments. These genes were distributed throughout the whole genome, although the introgression fragments were mainly located on chromosomes 4 and 5. One possible reason for this was that the introgressed genes might be involved in a complex regulatory network participating in KRN formation, and the change in these genes’ expression might result in transcriptional responses of related genes.

Previous studies have revealed some genes and pathways controlling KRN, such as some TF genes and RAMOSA, CLAVATA-WUSCHEL (CLV-WUS) and plant hormone signal pathways [9–20]. In our study, we also detected some important TF genes with significantly different expression between N531 and the ILs. For example, the SBP TF gene Zm00001d015410 was located in the common introgression fragments and meta-QTL on Chr. 5 [27]. As a type of plant-specific TF, SBP has been shown to play numerous important roles during plant growth and development, including flower and fruit development [35–38] and plant phase transition [39]. In maize, the mutants of the SBP TF genes *ub2* and *ub3* displayed a decrease in tassel branch number and an increase in KRN [19, 20]. Its negative regulatory role was confirmed by our results, because IL_A and IL_B had higher Zm00001d015410 expression and fewer KRNs. Eight MADS-box TF genes were also found and were all downregulated in the ILs. Previous studies have suggested that the MADS-box gene family can regulate the flowering time and inflorescence development. For example, *zea agamous-like1* (*zagl1*), which is a MADS-box transcription factor, can control the flowering time and reduce the kernel row number in maize [40]. Fifteen *AP2* genes were also found with significantly different expression between N531 and the ILs. In the *ub3* mutant, several AP2 domain genes were downregulated, which indicated the *AP2* genes might be involved in the inflorescence development and kernel row number processes [19]. Zm00001d017591, which encodes AP2-EREBP-transcription factor 10 protein, was located on Chr. 5 and downregulated in the ILs. These results provide valuable information for further research into cloning genes associated with the kernel row number.

Many previous studies have proven that regional association mapping can be used as an effective way to identify candidate genes or loci [24–27]. Here, we performed regional association mapping from 7 Mb to 156 Mb on Chr. 5 to identify SNPs significantly associated with KRN. When we combined these data with the RNA-Seq results, we found that three SNPs were located in three DEGs (Zm00001d013277, Zm00001d015310 and Zm00001d015377). Except for Zm00001d013277, the genes were all located in not only the common introgression fragment but also the meta-QTL on Chr. 5 [27]. Moreover, Zm00001d015310 was located within *qKRN5–1,* which was detected in the RIL population in our previous study [32]. This gene was dramatically downregulated, with the ILs exhibiting only 1% of the expression detected for N531. The only upregulated gene was Zm00001d015377, with approximately four-fold upregulated expression in the ILs. To validate the functions of the new genes, overexpression and knockdown of the genes to determine whether inflorescence development and KRN will change are necessary.

In this study, we combined transcriptome analysis and regional association mapping to mine new important genes controlling inflorescence development and KRN formation (Additional file 6: Figure S6). The DEGs among N531 and its ILs were identified by transcriptome analysis, and then the important candidate gene(s) were verified by regional association analysis. These results give us more information and new perspectives to identify critical regulators responsive to important steps in KRN formation.

## Conclusions

Kernel row number is a complex quantitative trait that is controlled by numerous QTLs with small effects in maize. In this study, a total of 2872 and 2428 DEGs were identified in N531 vs IL_A and N531 vs IL_B, respectively. Additionally, a total of 1252 DEGs were detected as overlapping DEGs, and 89 DEGs were located on the common introgression fragments. Combined with regional association mapping, we found that three SNPs which were located in three DEGs (Zm00001d013277, Zm00001d015310 and Zm00001d015377) associated with kernel row number. SNP1 was located in the Zm00001d013277 gene (which encodes a retrovirus-related pol polyprotein LINE-1 protein) and had two alleles (A/G) in the association panel, with the A allele being associated with a higher KRN (Fig. 5b, Table 1). For SNP48, which was located in the Zm00001d015310 gene (which encode a cystathionine beta-synthase (CBS) family protein), significant differences in KRN were found between its two alleles (C/T). Zm00001d015377 (which encodes a pentatricopeptide repeat-containing protein) contains SNP49 and the A allele of this SNP represented a higher KRN. These results confirmed that combining transcriptome analysis and regional association mapping was helpful for functional marker development and rapid determination of candidate genes or loci. The candidate genes identified in this study contribute to our understanding of the genetic architecture of the kernel row number in maize.

## Methods

### Plant materials

N531 is an elite maize inbred line, and SLN is a Chinese maize landrace. These lines are all conserved in the national germplasm bank of China. Previously, we used N531 with a KRN of 18–22 rows as the recurrent parent and SLN with a KRN of 4–6 rows as the donor parent to develop F_2:3_ and advanced backcross populations for QTL mapping. A major QTL named *qKRN5.04* was detected stably in different environments and generations [28, 29]. Through selection of a lower KRN and molecular marker-assisted selection (MAS) for *qKRN5.04* after multiple generations of backcrossing and selfing, we obtained two lines with a lower KRN from BC_5_F_7_, which were designated IL_A and IL_B (Additional file 7: Figure S7). In 2017, N531, SLN, IL_A and IL_B were grown in the experimental field of Chinese Academy of Agriculture Sciences in Beijing, China (39.97°N, 116.33°E). All the materials were planted in 3-m rows with 0.6 m between adjacent rows and 12 individual plants per row. The field management followed local practices. All plant materials in this study were conserved in our experimental lab. We declare that all plant materials in this study comply with the ‘Convention on the Trade in Endangered Species of Wild Fauna and Flora’.

### DNA extraction and genotyping

At the 11-leaf stage, the bulked fresh leaf tissues of N531, SLN, IL_A and IL_B were collected for DNA extraction according to the cetyltrimethylammonium bromide (CTAB) method [41]. All materials were genotyped using the MaizeSNP50 BeadChip, which is an Illumina BeadChip array of 56,110 maize SNPs developed from the B73 reference sequence [42]. We excluded SNPs with poor quality and selected polymorphic SNPs between the parents (N531 and SLN) to analyze the genomic composition of IL_A and IL_B.

### Sample preparation and RNA sequencing

The formation of the maize kernel row number is a key stage of maize inflorescence development and is influenced by many genes involved in inflorescence development. In this study, we select the 5-mm stage young ear for transcriptome analysis. In this stage, the IM, SPMs, SMs and FMs are present in the ears for N531, IL_A and IL_B (Fig. 1). Therefore, this strategy can help identify candidate genes that may affect the kernel row number in different meristems. At the 11-leaf stage, 5-mm immature ears of N531, IL_A and IL_B were sampled and immediately frozen in liquid nitrogen and then stored at − 80 °C. Each sample consisted of 10 ears pooled together and has two biological replications. Total RNA was extracted using TRIzol reagent (Invitrogen, CA, USA). cDNA library construction and sequencing of the transcriptome on the Illumina HiSeq 2000 platform (Illumina, CA, USA) were performed by Novogene (Beijing, China).

### Transcriptome data analysis

Clean reads were obtained by filtering raw reads containing adapters and more than 10% N (bases with uncertain information) as well as reads of low sequencing quality (more than 50% bases with Phred score ≤ 20). Those clean reads were mapped to the B73 reference genome (B73 RefGen_v3) (ftp://ftp.ensemblgenomes.org/pub/release-30/plants/fasta/zea_mays/dna/) using the TopHat2 software to count the total reads, mapped reads and uniquely mapped reads, and only the unique hits were kept for further analysis [43]. Gene expression levels were estimated with FPKM, and the Log_10_^(FPKM + 1)^ was used to calculate Pearson’s correlation coefficient between replications of different samples [44]. DESeq was used to identify genes with different expression levels [45]. The false discovery rate (FDR) was controlled by Benjamini and Hochberg’s approach [46]. Significantly DEGs were filtered by p_adj_ < 0.05. Gene ontology (GO) analysis of the DEGs was conducted, and significantly enriched GO terms were determined by a corrected *P-*value < 0.05 [47]. For the KEGG (Kyoto Encyclopedia of Genes and Genomes) analysis, a significantly enriched pathway was determined by a FDR < 0.05 with the Benjamini and Hochberg correction [46, 48].

### Regional association mapping

A maize natural variation panel consisting of 379 inbred lines was applied in this study to perform regional association mapping. We removed minor SNP states and selected SNP markers with minor allele frequencies > 0.05 from 7 Mb to 156 Mb on chromosome 5. A total of 28,763 SNPs were used for the regional association mapping analysis. The association analysis was conducted using the mixed linear model (MLM) after controlling for population structure (Q) and kinship (K) (MLM Q + K) in TASSEL V5.0 [49]. A significant marker-trait associations were declared at LOD > 2.5. The association mapping populations were planted in Beijing (39.48°N, 116.28°E) and arranged in a randomized complete block design. Each genotype was grown in a single row 3 m in length with 0.6 m between adjacent rows and 12 individual plants per row in a double-repeated format. The field management followed normal agricultural practices. After harvest, the KRN of every material was counted.

### Quantitative real-time RT-PCR

In total, eight common DEGs were randomly selected for quantitative real-time RT-PCR (qRT-PCR). The first strands of the cDNA were synthesized from RNA using *TransScript* – Uni One-Step gDNA Removal and cDNA Synthesis SuperMix (TransGen Biotech, Beijing, China). Quantitative RT-PCR was carried out in triplicate for each sample using the SYBR Premix Ex Taq (Takara, Japan) on a 7500 Real-Time PCR System (Applied Biosystems, CA, USA). Each 20-μl PCR contained 10 μl of 2× SYBR Premix Ex Taq (Takara, Japan), 8 μl of ddH_2_O, 0.8 μl of cDNA, 0.4 μl of Dye II, 0.4 μl of forward primer and 0.4 μl of reverse primer. The thermal cycling conditions were as follows: 95 °C for 30 s at holding stage, 95 °C for 5 s, 60 °C for 34 s at cycling stage, 95 °C for 15 s, 60 °C for 1 min, 95 °C for 30 s, and 60 °C for 15 s at melt curve stage. The expression levels of genes in the samples were normalized using maize *GAPDH,* and the relative expression levels were calculated using the 2^-△△Ct^ method [50]. The primer sequences used in this step were designed using Primer 5.0 and are listed in Additional file 18: Table S11.

## Additional files

Additional file 1: **Figure S1.** Correlation analysis of the transcriptomes of each replicate. The correlation of each replicate transcriptome was analyzed by Log10 (FPKM + 1). The correlations among N531, IL_A and IL_B were plotted. (TIF 179 kb)

Additional file 2: **Figure S2.** Gene function analysis. (a) Gene ontology (GO) enrichment of DEGs for biological process, cellular component and molecular function. All GO terms are significant at FDR < 0.01. (b) KEGG pathway enrichment of DEGs. The scatter diagram shows the most significant 20 pathway terms. The color of the dots represents different q values, and the size of the dots represents the number of DEGs in this pathway term. (TIF 2104 kb)

Additional file 3: **Figure S3.** Major QTLs detected for KRN in the RIL population in Henan in 2009 [32]. (TIF 81 kb)

Additional file 4: **Figure S4.** Validation of the RNA-Seq results via qRT-PCR. (a) Four genes randomly selected from upregulated DEGs and four genes randomly selected from downregulated DEGs. For each plot, the left columns represent the results in quantitative real-time RT-PCR (qRT-PCR), and the right columns represent FPKM in RNA-Seq. (b) The log2-transformed qRT-PCR expression data are plotted against log2-transformed RNA-Seq data and fit to a linear regression. (TIF 289 kb)

Additional file 5: **Figure S5.** Expression of three candidate genes in different tissues, including the root, stem and leaf, during the three-leaf stage. (TIF 67 kb)

Additional file 6: **Figure S6.** Diagram of the strategy used to mine the proposed candidate genes using a combination of different experiments and methods. (TIF 94 kb)

Additional file 7: **Figure S7.** Diagram of the creation process for the introgression lines (IL_A and IL_B). (TIF 51 kb)

Additional file 8: **Table S1.** Distribution of different genotypes in IL_A and IL_B. (XLSX 11 kb)

Additional file 9: **Table S2.** Reads statistics for N531 and its introgression lines. (XLSX 10 kb)

Additional file 10: **Table S3.** List of the DEGs between N531 and IL_A. (XLSX 170 kb)

Additional file 11: **Table S4.** List of the DEGs between N531 and IL_B. (XLSX 145 kb)

Additional file 12: **Table S5.** List of the DEGs between N531 vs IL_A and N531 vs IL_B. (XLSX 183 kb)

Additional file 13: **Table S6.** List of the DEGs between IL_A and IL_B. (XLSX 107 kb)

Additional file 14: **Table S7.** List of the DEGs among IL_A, IL_B and N531. (XLSX 17 kb)

Additional file 15: **Table S8.** List of the DEGs located in the common introgression fragments between N531 vs IL_A and N531 vs IL_B. (XLSX 21 kb)

Additional file 16: **Table S9.** Genes that may be involved in maize inflorescence development. (XLSX 15 kb)

Additional file 17: **Table S10.** Summary of SNPs associated with KRN. (XLSX 13 kb)

Additional file 18: **Table S11.** Primers used in qRT-PCR. (XLSX 9 kb)

## Abbreviations

DEGs: Differentially expressed genes
FDR: False discovery rate
FMs: Floral meristems
FPKM: Expected number of Fragments Per Kilobase of transcript sequence per Millions base pairs sequenced
GO: Gene Ontology
ILs: Introgression lines
IM: Inflorescence meristem
KEGG: Kyoto Encyclopedia of Genes and Genomes
KRN: Kernel row number
MLM: Mixed linear model
QTL: Quantitative trait locus
SBP: SQUAMOSA promoter-binding protein
SMs: Spikelet meristems
SNP: Single nucleotide polymorphism
SPMs: Spikelet pair meristems
TFs: Transcription factors
