# Supplementary figures and images for: Candidate loci for the kernel row number in maize revealed by a combination of transcriptome analysis and regional association mapping
Source: BMC Plant Biol. 2019 May 16;19:201. doi: 10.1186/s12870-019-1811-1 (PMC6521486; doi:10.1186/s12870-019-1811-1)

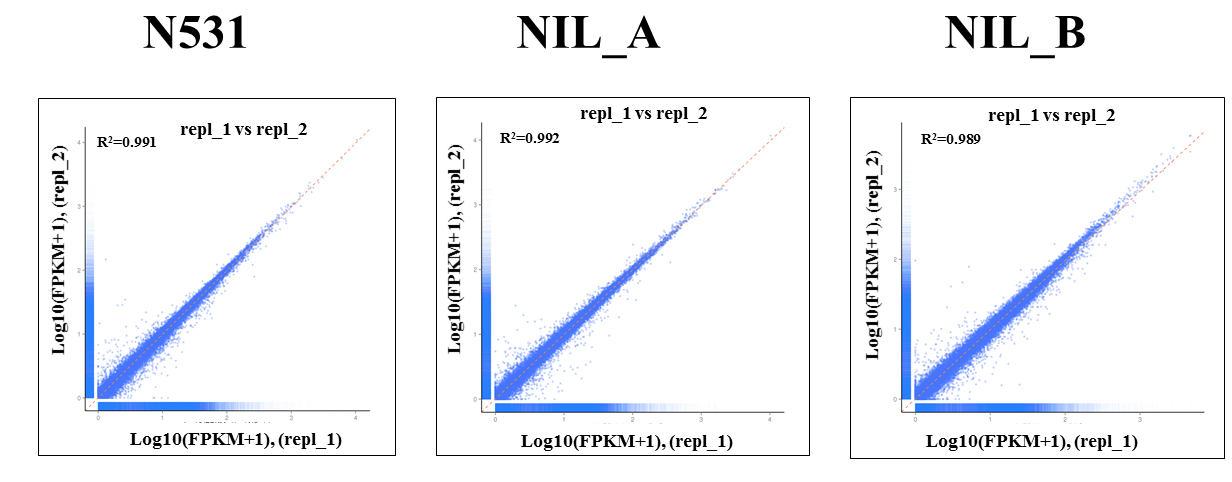

Supplement: Supplementary file 1 — Figure S1. Correlation analysis of the transcriptomes of each replicate. The correlation of each replicate transcriptome was analyzed by Log10 (FPKM + 1). The correlations among N531, IL_A and IL_B were plotted. (TIF 179 kb) [file 12870_2019_1811_MOESM1_ESM.tif]

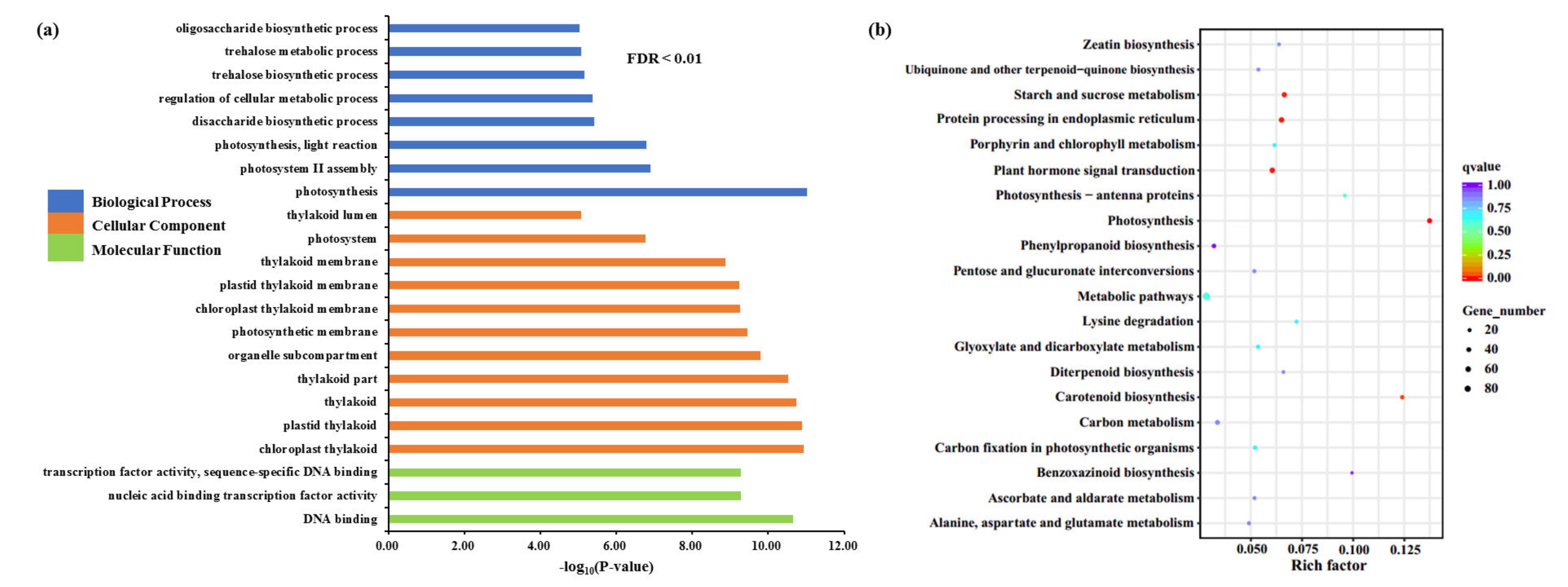

Supplement: Supplementary file 2 — Figure S2. Gene function analysis. (a) Gene ontology (GO) enrichment of DEGs for biological process, cellular component and molecular function. All GO terms are significant at FDR < 0.01. (b) KEGG pathway enrichment of DEGs. The scatter diagram shows the most significant 20 pathway terms. The color of the dots represents different q values, and the size of the dots represents the number of DEGs in this pathway term. (TIF 2104 kb) [file 12870_2019_1811_MOESM2_ESM.tif]

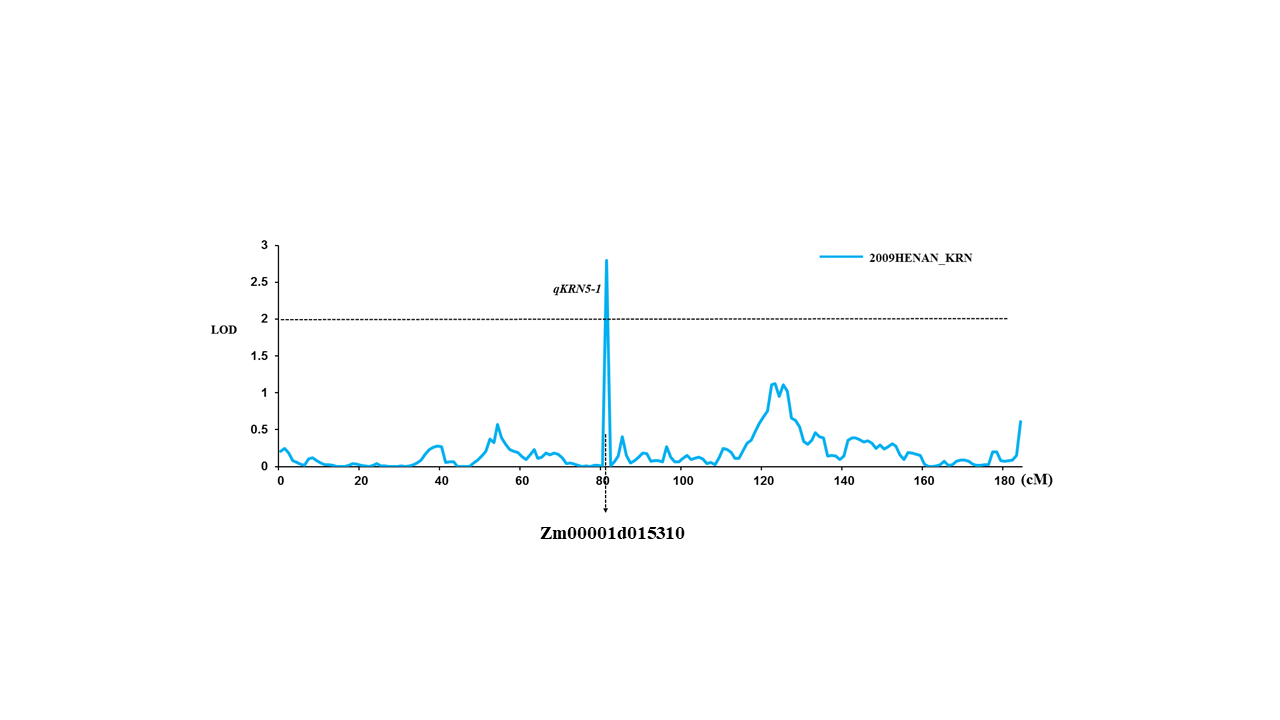

Supplement: Supplementary file 3 — Figure S3. Major QTLs detected for KRN in the RIL population in Henan in 2009 [32]. (TIF 81 kb) [file 12870_2019_1811_MOESM3_ESM.tif]

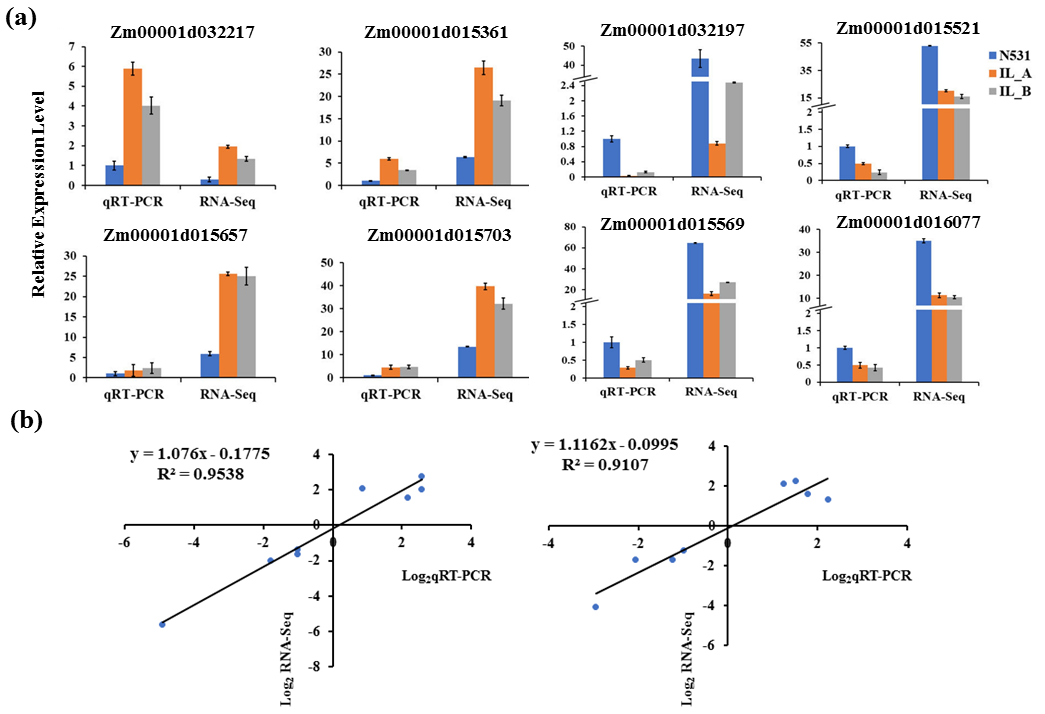

Supplement: Supplementary file 4 — Figure S4. Validation of the RNA-Seq results via qRT-PCR. (a) Four genes randomly selected from upregulated DEGs and four genes randomly selected from downregulated DEGs. For each plot, the left columns represent the results in quantitative real-time RT-PCR (qRT-PCR), and the right columns represent FPKM in RNA-Seq. (b) The log2-transformed qRT-PCR expression data are plotted against log2-transformed RNA-Seq data and fit to a linear regression. (TIF 289 kb) [file 12870_2019_1811_MOESM4_ESM.tif]

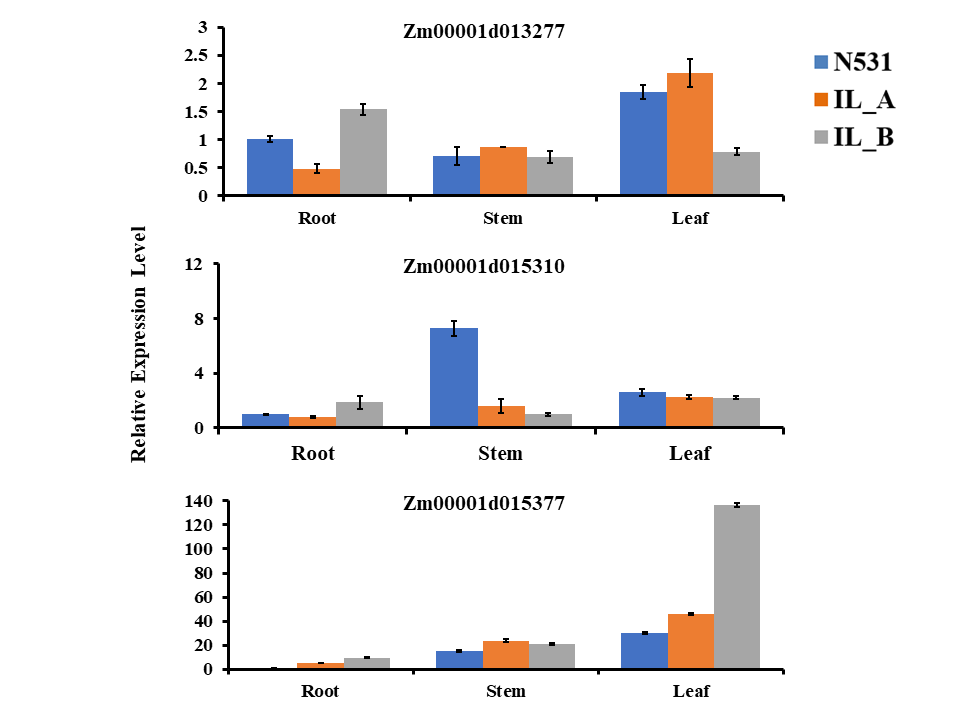

Supplement: Supplementary file 5 — Figure S5. Expression of three candidate genes in different tissues, including the root, stem and leaf, during the three-leaf stage. (TIF 67 kb) [file 12870_2019_1811_MOESM5_ESM.tif]

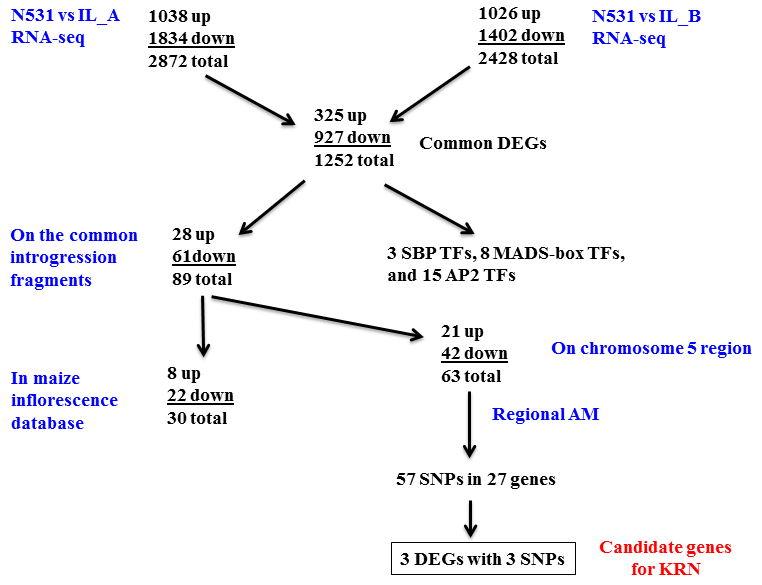

Supplement: Supplementary file 6 — Figure S6. Diagram of the strategy used to mine the proposed candidate genes using a combination of different experiments and methods. (TIF 94 kb) [file 12870_2019_1811_MOESM6_ESM.tif]

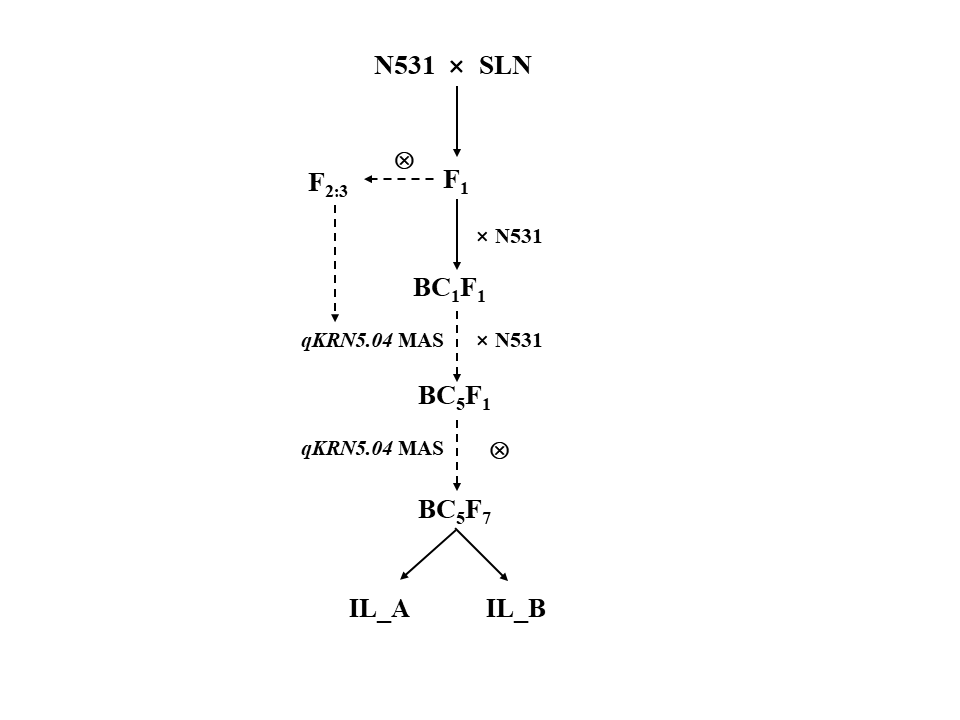

Supplement: Supplementary file 7 — Figure S7. Diagram of the creation process for the introgression lines (IL_A and IL_B). (TIF 51 kb) [file 12870_2019_1811_MOESM7_ESM.tif]
